# Similar dietary regulation of IGF-1- and IGF-binding proteins by animal and plant protein in subjects with type 2 diabetes

**DOI:** 10.1007/s00394-021-02518-y

**Published:** 2021-03-08

**Authors:** Rita Schüler, Mariya Markova, Martin A. Osterhoff, Ayman Arafat, Olga Pivovarova, Jürgen Machann, Johannes Hierholzer, Silke Hornemann, Sascha Rohn, Andreas F. H. Pfeiffer

**Affiliations:** 1 Department of Clinical Nutrition, German Institute of Human Nutrition Potsdam-Rehbrücke (DIfE), 14558 Nuthetal, Germany; 2 German Center for Diabetes Research (DZD), 85764 München-Neuherberg, Germany; 3 Department of Endocrinology, Diabetes and Nutrition, Charité-Universitätsmedizin Berlin, Campus Benjamin Franklin, 12200 Berlin, Germany; 4 Institute of Diabetes Research and Metabolic Diseases (IDM) of the Helmholtz Center Munich at the University of Tübingen, Tübingen, Germany; 5 Section of Experimental Radiology, University Hospital Tübingen, Tübingen, Germany; 6 Diagnostic and Interventional Radiology, Klinikum Ernst von Bergmann, Academic Teaching Hospital, Charité-Universitätsmedizin Berlin, Potsdam, Germany; 7 Institute of Food Chemistry, Hamburg School of Food Science, University of Hamburg, Hamburg, Germany; 8 Department Endocrinology and Metabolism, Charité Universitätsmedizin Berlin, Campus Benjamin Franklin, Hindenburgdamm 30, 12203 Berlin, Germany

**Keywords:** Protein intake, Growth hormone, Insulin-like growth factor-1, IGF-binding protein-1, IGF-binding protein-2, Plant protein, Animal protein

## Abstract

Increased animal but not plant protein intake has been associated with increased mortality in epidemiological studies in humans and with reduced lifespan in animal species. Protein intake increases the activity of the IGF-1 system which may provide a link to reduced lifespan. We, therefore, compared the effects of animal versus plant protein intake on circulating levels of IGF-1 and the IGF-binding proteins (IGFBP)-1 and IGFBP-2 over a 6-week period. Thirty seven participants with type 2 diabetes consumed isocaloric diets composed of either 30% energy (EN) animal or plant protein, 30% EN fat and 40% EN carbohydrates for 6 weeks. The participants were clinically phenotyped before and at the end of the study. Both diets induced similar and significant increases of IGF-1 which was unaffected by the different amino acid compositions of plant and animal protein. Despite improvements of insulin sensitivity and major reductions of liver fat, IGFBP2 decreased with both diets while IGFBP-1 was not altered. We conclude that animal and plant protein similarly increase IGF-1 bioavailability while improving metabolic parameters and may be regarded as equivalent in this regard.

## Introduction

The growth hormone/insulin-like growth factor-1 (IGF-1) system is highly interrelated with the insulin signaling pathway and function [1]. Reduced activation of insulin and IGF-1-pathways has been linked to longevity and a reduction of cancer, but it remains unclear whether this is linked to reduced IGF-1 pathway activity or to improved insulin sensitivity [2–5]. High protein intake increases circulating IGF-1 and reduces lifespan in C. elegans, flies and some strains of mice [2, 4]. A recent analysis of the NHANES III-study suggested that older people—in contrast to younger subjects—show improved survival with high protein intake suggesting an age-dependent response [6]. Moreover, plant protein intake in contrast to animal protein did not influence the association between protein intake and mortality [6]. In fact, epidemiological data suggest reduced mortality and cardiovascular events with higher plant protein intake [7]. The amino acid composition of protein therefore appears to determine responses [8], but it is not known, whether animal and plant proteins differ in their ability to increase IGF-1. Higher levels of IGF-1 were associated with improved muscle and bone mass [3, 9] and higher animal protein intakes were associated with reduced risks of osteoporotic fractures [10, 11]. Moreover, higher levels of IGF-1 may be associated with reduced risk of cardiovascular disease [12, 13]. Elevated levels of IGF-1 were linked to reduced risk of type 2 diabetes [14] in some studies while others observed neutral effects [3].

Less than 1% of circulating IGF-1 is free and thus bioactive. The bioactivity of IGF-1 is regulated by IGF-binding proteins (IGFBP). In particular IGFBP1 and IGFBP2 exert inhibitory actions on IGF-1 bioactivity and are strongly regulated by insulin and food intake [15–17]. A decrease of IGF-1 and an increase of IGFBP1 was moreover associated with increased risk of developing cardiac failure in older people with type 2 diabetes [18]. IGFBP-1 is acutely down-regulated by insulin and correlates closely with insulin sensitivity. Obese subjects and people with type 2 diabetes had lower levels of IGFBP1 and weight loss, as well as exercise increased levels of IGFBP1 [16, 17]. IGFBP2 was reported to correlate with increased intake of protein in epidemiological studies [19–21]. Humans with impaired glucose metabolism had reduced levels of IGFBP2 and IGFBP2 positively correlated with insulin sensitivity [16]. IGFBP2 moreover inhibited the differentiation of visceral but not subcutaneous adipocytes and was associated with a reduction of hepatic fat content in leptin-deficient mouse models [17].

The ideal amount and type of dietary protein intake is highly controversial at present due to its associations with life expectancy and disease risks in animal models and humans [2, 22]. We, therefore, evaluated the effects of animal versus plant protein-enriched diets (30% protein, 40% carbohydrates, 30% fat) in older patients with type 2 diabetes on circulating IGF-1, IGFBP1 and IGFBP2 levels. We particularly focused on the question whether animal and plant protein differentially alter the IGF-system in view of their different associations with disease risks which were proposed to be mediated by the IGF-system [2, 22]. We moreover assessed associations of changes of the IGF-system with changes of insulin sensitivity and anthropometry, particularly abdominal and subcutaneous fat mass, muscle mass and hepatic fat content in response to protein-rich diets.

## Methods

### LeguAn study

The LeguAn study protocol was approved by the ethics committee of the Charité-Universitätsmedizin Berlin and conducted in accordance with the principles of the Helsinki Declaration of 1975, as revised in 2000. All participants gave written informed consent prior to the study. A detailed description of the study design was published recently [23, 24]. The LeguAn study was registered with ClinicalTrials.gov: NCT02402985.

37 participants (24 males and 13 females) with type 2 diabetes (mean HbA1c 7.0 ± 0.1%) and a mean age of 64 ± 1 years were randomized according to weight, age, sex, HbA1c and medication as described in detail [25] and received an isocaloric high-protein diet (30% protein, 40% carbohydrates and 30% fat) for 6 weeks. Blood samples were collected after an overnight fast before and after the high-protein dietary intervention.

### Serum concentrations of IGF-1 and IGFBPs

Concentrations of serum IGF-1, IGFBP1 and IGFBP2 were measured using ELISA (Mediagnost, Reutlingen, Germany).

### Statistical analysis

Variables were tested for normal distribution using the Kolmogorov–Smirnov test. One-way or repeated measures ANOVA followed by Bonferroni post hoc test was used for statistical comparison of mean values of continuous data. Pearson’s and Spearman’s rank correlation coefficients were used for correlation analysis for variables with normal and skewed distribution, respectively. Unless otherwise stated data are reported as mean ± SEM. Statistical significance level was designated at *P* < 0.05. Statistical analysis was performed using SPSS 20.0 (IBM, Chicago, IL, USA).

## Results

A total of 37 overweight to moderately obese subjects with T2DM completed the study. Patients were on average 64 years old and under oral antidiabetic treatment with HbA1c values of 7%. At baseline, the participants consumed diets which contained 42% carbohydrates, 17% protein and 41% fat. They were randomized to receive either an animal protein (AP) rich or a plant protein (PP)-rich diet. During the intervention, the protein intake was increased to 30% of energy intake and exchanged against fat intake in both groups as previously described in detail [23, 24]. The diets were partly supplied and the protein intake was monitored by determination of urea in the blood and urine which increased by about 30%. The plant protein diets contained pea protein-enriched noodles, bread and a pea mash to achieve the high plant protein intake over 6 weeks. The amino acid composition of the nutrition was calculated from dietary protocols [25] and plasma levels were determined after representative meals [26] which showed higher concentrations of the branched chain amino acids, tryptophane and methionine in the animal protein group while asparagine and arginine were higher in plant protein group. Fasting levels of amino acids did not differ between the diets [23].

The high-protein diet induced a highly significant increase in IGF-1 by about 14% overall (Table 1). Quite unexpectedly this increase was seen in both groups, i.e. with high plant protein and with high animal protein without significant differences between the groups (*p* = 0.639, Table 2). IGFBP1 concentrations did not change with the diets neither overall nor in the AP and PP groups, respectively (*p* = 0.361, *p* = 0.215 and *p* = 0.968). In contrast, IGFBP2 levels decreased significantly overall by 12% (Table 1). Stratified by the protein source, this decrease was not significant neither in the AP nor in the PP group although a similar tendency was observed in both groups.

**Table 1:** Effects of HP diet on IGF-1 and IGFBPs concentrations

	0 weeks	6 weeks	*p*
Glucose (mmol/l)	9.56 ± 1.64	8.99 ± 1.90	**5.2 × 10**^**–4**^
Insulin (mU/l)	9.39 ± 6.43	8.73 ± 6.49	0.145
HOMA-IR	4.08 ± 3.07	3.42 ± 2.38	**0.016**
IGF-1 (µg/l)	183.6 ± 8.2	209.2 ± 7.8	**4.0 × 10**^**–6**^
IGFBP-1 (µg/l)	5.5 ± 0.6	5.5 ± 1.0	0.361
IGFBP-2 (µg/l)	260.3 ± 25.0	228.2 ± 17.8	**0.015**

Values are shown as mean ± SD. (Wilcoxon)

**Table 2:** Effects of HP diet on IGF-1 and IGFBPs concentrations stratified by protein source

	AP			PP		
	0 weeks	6 weeks	*p*	0 weeks	6 weeks	*p*
Glucose (mmol/l)	9.64 ± 1.81	8.61 ± 1.51	**0.006**	9.48 ± 1.49	9.35 ± 2.19	0.039
Insulin (mU/l)	10.07 ± 7.18	8.31 ± 5.40	0.109	8.74 ± 5.75	9.12 ± 7.50	0.536
HOMA-IR	4.45 ± 3.71	3.15 ± 2.09	**0.032**	3.71 ± 2.33	3.67 ± 2.65	0.651
IGF-1 (µg/l)	183.3 ± 13.0	210.8 ± 12.6	**1.1 × 10**^**–4**^	183.8 ± 10.5	207.7 ± 9.7	**3.0 × 10**^**–4**^
IGFBP-1 (µg/l)	5.3 ± 0.9	4.8 ± 1.3	0.215	5.7 ± 0.9	6.1 ± 1.6	0.968
IGFBP-2 (µg/l)	229.8 ± 26.7	206.7 ± 20.6	0.078	289.1 ± 41.3	248.5 ± 28.4	0.117

IGF-1 levels significantly correlated with IGFBP2 levels at baseline (ρ = 0.456, *p* = 0.005). IGFBP1 and IGFBP2 levels were significantly correlated before and after the high-protein diet (ρ = 0.369, *p* = 0.025 and ρ = 0.435, *p* = 0.007). Baseline levels of IGF-1 modestly negatively correlated with BMI but not with other anthropometric parameters. Furthermore, IGF-1 was strongly negatively correlated with hepatic fat content determined by MRI spectroscopy before and after the high-protein diet (Table 3). IGFBP1 showed highly negative correlations with anthropometric measures including BMI, waist circumference and WHR and moderate negative correlations with fasting insulin and insulin sensitivity as determined by HOMA. IGFBP2 was highly negatively correlated with fasting insulin and HOMA and with hepatic fat content in addition to BMI and waist circumference (Table 3).

**Table 3:** Correlation analysis of IGF-1 and IGFBPs with anthropometric and biochemical parameters before and after high-protein diet

		BMI	Waist	WHR	VAT(l)	IHL	Insulin	HOMA-IR
IGF-1	0 weeks	– 0.345*				– 0.530**		
	6 weeks					– 0.587***		
IGFBP1	0 weeks	– 0.437**	– 0.564***	– 0.502**			– 0.358*	– 0.348*
	6 weeks	– 0.427**	– 0.578***		– 0.520**		– 0.496**	– 0.559***
IGFBP2	0 weeks	– 0.570***	– 0.542***			– 0.548**	– 0.579***	– 0.612***
	6 weeks	– 0.598***	– 0.567***		– 0.413*	– 0.501**	– 0.620***	– 0.707***

Correlation of IGF-1 and IGFBPs with anthropometric measures and metabolic parameters. Only significant correlations (Spearman’s rank correlation coefficients) are presented

*P < 0.05, **P < 0.01 and ***P < 0.001

The highly negative correlation of IGF-1 and of IGFBP2 with the hepatic fat content was preserved despite a reduction of hepatic fat content by 47% after 6 weeks of high-protein diet. Similarly, the correlation of IGFBP1 and IGFBP2 with insulin sensitivity and anthropometric variables became somewhat stronger after 6 weeks (Table 3).

As recently published, the high-protein diet–although without caloric restriction—resulted in a strong reduction of hepatic fat content and an increase of lean body mass with a reduction of body fat [23]. Notably, the visceral fat volume was negatively correlated with IGFBP1 and IGFBP2 after the dietary protein intervention but not before which aligns well with the inhibition of visceral fat cell proliferation by IGFBP2 in experimental models [17].

## Discussion

In the LEGUAN study, we addressed the question whether the protein source in a high-protein diet, animal- vs. plant-based protein, is a relevant determinant of circulating IGF-1, IGFBP1 and 2 levels in participants with type 2 diabetes.

The most striking finding is that plant protein induces similar increases of IGF-1 as animal protein does. In view of the associations of PP with reductions in overall and cardiovascular mortality as compared to AP this is highly relevant [7]. It provides an argument against a role of IGF-1 in mediating the negative effects of high animal protein intake. Indeed the literature on this topic is rather inconclusive at present [27]. Accordingly, our results suggest that differences between plant vs. animal protein intake and cancer or cardiovascular risk do not arise from their effects on components of the IGF-1-system but protective properties of other components present in plant-based diets. Notably, most epidemiological studies do not account for confounding due to differences in lifestyle of meat consumers versus vegetarians. Recent large population-based cohort studies extensively correcting for confounding did not find differences in mortality between vegetarians and non-vegetarians [28, 29].

Vegan diet was associated with lower IGF-1 and higher IGFBP1 and IGFBP2 levels in cross-sectional studies compared to vegetarian and meat-containing diets. Low levels of IGF-1 were associated with increased risk for angiographically confirmed coronary heart disease in cross-sectional [30] and prospective studies [31] and predicted death therefrom[32]. This may be explained by the rather potent effects of IGF-1 on cardiac remodeling and endothelial as well as smooth muscle cells [33, 34]. IGF-1 prevents age-related decreases in cardiomyocyte number, decreased synthesis of contractile proteins and fibrosis [35]. Therefore, changes induced by low-protein diets in these components of the IGF-system appear to be unfavorable and outcomes from studies in short lived animals which are little affected by cardiovascular disease should not be translated to humans.

A further unexpected finding of our study was that IGFBP2 levels decreased upon high protein intake. Elevated levels of IGFBP2 were associated with death from ischemic heart disease [36] and were also a biomarker of increased mortality in elderly subjects [37]. On the other hand, IGFBP2 decreased liver fat and improved metabolic markers upon overexpression in mice [38] and was associated with a more favorable metabolic profile in humans [37]. We observed similar inverse associations of IGFBP2 with visceral adipose tissue, liver fat and insulin levels and insulin sensitivity in our study. In fact, the inverse associations with visceral adipose tissue and liver fat became much more pronounced after 6 weeks of high-protein diet, despite the decrease of IGFBP2 (Table 3). We therefore interpret the reduction of IGFBP2 as an advantage which will enhance the increase of bioactive IGF-1 particularly in the extravascular compartment [27].

The observation that IGFBP1 was not altered is noteworthy since increases of IGFBP-1 were reported to associate with the beneficial effects of protein restriction in animal studies. In humans, increased levels of IGFBP-1 not only predicted heart failure in a prospective study after myocardial infarction but also overall mortality over 8 years [39]. IGFBP1 was also elevated in patients with severe coronary heart disease in people with [40] and without [41] type 2 diabetes. IGFBP1 is strongly regulated by insulin and decreased in subjects with insulin resistance [16] and was inversely correlated with insulin levels, waist circumference and abdominal fat content in this study [40].

It has been postulated that dietary intake of essential amino acids is a determining factor of IGF-1 levels. Allen and coworkers postulated that the lower intake of protein high in essential amino acids may account for the difference in IGF-1 and IGFBP concentrations between diets [42]. However, these studies were cross-sectional. Plant protein differed in our study from the animal protein by a lower content of leucine and methionine and a higher content of arginine and asparagine. These intakes altered postprandial but not fasting levels of these plasma amino acids as we previously reported [26]. Since both diets had similar effects on IGF-1 and IGFBP1 and 2, our data do not support an important effect of the protein composition but rather suggest that the amount of protein determines the response in case of high-protein diets. This may, however, not apply to low-protein diets were the differences in amino acid composition may lead to specific deficiencies and thereby induce amino acid driven-specific hormonal responses as recently reported for FGF21 and IGF-1 [43]. Nevertheless, levels of IGF-1 were primarily related to the nitrogen balance and less to deficiency of specific amino acids.

In summary, our data show that animal and plant proteins exert similar effects on IGF-1, IGFBP2 and 3. The increases in IGF-1 and moderate decreases in IGFBP2 are likely to exert positive effects on cardiovascular and bone health and were associated with significant improvements of metabolic risk markers such as liver fat content, insulin sensitivity and visceral and subcutaneous fat content. Therefore, the current study provides arguments for an increased protein intake in the context of a healthy diet regarding fat and plant food intake in participants with type 2 diabetes and an age above 60 years.
